# Mechanisms of Electroacupuncture-Induced Analgesia on Neuropathic Pain in Animal Model

**DOI:** 10.1155/2013/436913

**Published:** 2013-07-31

**Authors:** Woojin Kim, Sun Kwang Kim, Byung-Il Min

**Affiliations:** ^1^Department of East-West Medicine, Graduate School, Kyung Hee University, Seoul 130-701, Republic of Korea; ^2^Department of Physiology, College of Korean Medicine, Kyung Hee University, Seoul 130-701, Republic of Korea; ^3^Department of Physiology, College of Medicine, Kyung Hee University, Seoul 130-701, Republic of Korea

## Abstract

Neuropathic pain remains as one of the most difficult clinical pain syndromes to treat. Electroacupuncture (EA), involving endogenous opioids and neurotransmitters in the central nervous system (CNS), is reported to be clinically efficacious in various fields of pain. Although multiple experimental articles were conducted to assess the effect of EA-induced analgesia, no review has been published to assess the efficacy and clarify the mechanism of EA on neuropathic pain. To this aim, this study was firstly designed to evaluate the EA-induced analgesic effect on neuropathic pain and secondly to guide and help future efforts to advance the neuropathic pain treatment. For this purpose, articles referring to the analgesic effect of acupuncture on neuropathic pain and particularly the work performed in our own laboratory were analyzed. Based on the articles reviewed, the role of spinal opioidergic, adrenergic, serotonergic, cholinergic, and GABAergic receptors in the mechanism of EA-induced analgesia was studied. The results of this research demonstrate that *μ* and *δ* opioid receptors, **α**_2_-adrenoreceptors, 5-HT_1A_ and 5-HT_3_ serotonergic receptors, M_1_ muscarinic receptors, and GABA_A_ and GABA_B_ GABAergic receptors are involved in the mechanisms of EA-induced analgesia on neuropathic pain.

## 1. Introduction

Acupuncture has been a widely used method in traditional medicine in East Asia for thousands of years. Since its introduction to western countries in the 1970s, the global interest in acupuncture has increased, and significant evidence supports acupuncture as a useful tool for treating a diverse spectrum of diseases. In fact, more than 40 disorders have been endorsed by the World Health Organization (WHO) as conditions that can benefit from acupuncture treatment [1]. Among these disorders, pain is known to be particularly sensitive to acupuncture and has been a compelling field for research. In a total of 3,975 acupuncture research articles published from 1991 to 2009, 1647 (41%) focus on pain and analgesia [2].

Multiple theories of pain control mechanisms such as gate-control theory [3], spinal segmental mechanism [4], endogenous opioid system [5], descending noradrenergic and serotonergic systems [6], and diffuse noxious inhibitory control [7] have been investigated over the last several decades to clarify the mechanism of acupuncture and EA. Acupuncture is now proven to be clinically efficacious in various fields of pain, such as, lower back pain [8], chronic knee pain [9], and chronic headache [10], and a recent meta-analysis also demonstrates the effect of acupuncture on different types of chronic pain [11]. EA is a modified acupuncture technique that, as its name implies, utilizes electrical stimulation, and its analgesic effect on different types of acute pains and persistent inflammatory pains has appeared in both rodents and humans [12–14].

According to the NeuPSIG (Special Interest Group on Neuropathic Pain), neuropathic pain is defined as “pain arising as a direct consequence of a lesion or disease affecting the somatosensory system” [15]. It is often reported as having a lancinating or continuous burning character and is frequently associated with the appearance of abnormal sensory signs such as allodynia (pain as a result of a stimulus which normally does not provoke pain) or hyperalgesia (an increased response to a stimulus which is normally painful) [16]. The underlying mechanisms are complex and appear to involve both peripheral and central components of the nervous system [17].

 The spectrum of neuropathic pain covers a variety of disease states and presents itself in the clinic through a variety of symptoms, namely, lumbar radiculopathy (lower back pain caused by disk compression or herniation), spinal cord injury, phantom pain, diabetic neuropathy, postherpetic neuralgia, and in some patients, fibromyalgia, and cancer-related pain [18].

It is estimated that neuropathic pain affects over 26 million patients worldwide, resulting in a worldwide healthcare cost of over $3 billion per year, with a significant portion of this money paid to drug therapies that originally were developed for other medical conditions [19]. 

Current pharmacological treatment for neuropathic pain typically will include some combination of agents from several of the following drug classes: opioids, tricyclic antidepressants, anticonvulsant agents, or nonsteroidal anti-inflammatory drugs (NSAIDs)/analgesics. Ironically, even with such an impressive arsenal of powerful drugs, these approaches only provide an approximate 30–50% reduction of pain in about 50% of patients. In addition, there are various side effects associated with these drugs [20, 21].

These results underscore the importance of considering a complementary and alternative neuropathic pain treatment. Previously, several clinical studies have shown the effectiveness of EA on various neuropathic pain diseases such as neuropathic pain of malignancy [22], diabetic neuropathy [23, 24], phantom limb pain [25, 26], and below-level central neuropathic pain [27]. However, although multiple reviews exist on the analgesic mechanisms of EA, no previous review has been published on the effect of EA in neuropathic pain, and still the mechanism that lies behind it remains unclear.

For many years, and since the publishing of the first article of Hwang et al. [28] on neuropathic pain published from our laboratory, our research has focused on clarifying the mechanisms of EA on neuropathic pain, and different experimental designs were used to understand the analgesic effect of EA in neuropathic pain rats. So far, the mechanisms of EA on spinal endogenous opioidergic [28–31] adrenergic and serotonergic [32], cholinergic [33, 34], and GABAergic [35] systems have been clarified as a result of these studies, and efforts to clarify further mechanisms are on their way [36].

To guide future efforts in the advancement of neuropathic pain treatment, we believed that a timely review was important. In this review, based on the articles published in our laboratory, we will proceed to expand on clarification of the effect of EA on neuropathic pain and quantify its mechanism.

## 2. Endogenous Opioids and Descending Inhibitory System

Since 1970, the mechanism of acupuncture analgesia has been broadly investigated, and numerous pieces of evidence demonstrated that acupuncture analgesia is mediated via neuronal mechanisms correlated with the central nervous system (CNS) [6, 37, 38]. The most recognized mechanisms are endogenous opiates mechanisms [5, 39] and descending inhibitory mechanisms [6]. Endogenous opioids are known to be mediated through its *μ*, *δ*, *κ* receptors and descending inhibitory pathway through its monoaminergic neurotransmitters and their receptors. In this review, to clarify the mechanism of EA in neuropathic pain, we have focused on the neurotransmitters receptors present in the CNS, especially in the spinal level.

### 2.1. Opioidergic Receptors

Ever since the publication of the article by Arner and Meyerson in 1988 titled “lack of an effect of opioids on neuropathic and idiopathic forms of pain” [40], multiple studies have been published supporting the efficacy of opioids for neuropathic pain, and it is now known that opioids can clearly provide effective analgesia for neuropathic pain [41, 42]. Endogenous opioids are involved in both ascending and descending parts of the inhibition pathway. In the ascending portion, all three receptors (*μ*, *δ*, *κ*) play a part, but only *μ* and *δ* receptors are responsible in the descending portion [43].

The involvement of opioid receptors in mediating acupuncture analgesia is demonstrated in several articles. Han reported that EA analgesia is mediated by enkephalin, *β*-endorphin, endomorphin, and dynorphin released in the CNS and that *μ*, *δ*, *κ* opioid receptors are involved in the mechanisms [39, 44]. David Mayer clarified the role of endogenous opioid in the mechanisms proving that the analgesic effect of acupuncture was prevented or reversed by the opioid receptors blocker naloxone [45, 46].

To determine whether the EA analgesic effect is mediated by endogenous opioid in the rat model of neuropathic pain, Hwang et al. conducted an experiment by injecting opioid antagonist naloxone intraperitoneally 20 min before the EA in rat specimens [28]. The EA was applied at Houxi acupoint (SI3), and mechanical allodynia was assessed by a normally innocuous stimulation of the tail with the Von Frey Hair. An abrupt tail movement of more than 0.5 cm was considered to be an abnormal response attributed to mechanical allodynia. The results show that the antiallodynic effect of EA was reversed by intraperitoneal injected naloxone but not through normal saline. Further experimentation with intraperitoneal morphine also demonstrates that mechanical allodynia was relieved in a dose-dependent manner. The result reports that a higher dose (1.5 mg/kg) of morphine relieves more effectively the signs of mechanical allodynia than a lower dose (0.5 mg/kg) and that EA with 1.5 mg/kg of morphine induced a slightly more antiallodynic effect. These results are consistent with the previous results of Mayer and Omana [46, 47].

In addition, through cDNA microarray analysis and dot-blot analysis, Ko et al. identified the opioid signaling events involved in neuropathic pain [31]. This data suggest that the opioid receptor probably plays an important role in the development of neuropathic pain and the analgesic effects of EA.

Furthermore, Kim et al. [29] conducted an experiment to clarify which opioidergic receptors are involved in the relieving effect of EA on mechanical allodynia in the spinal cord. Selective *μ* (*β*-FNA), *δ* (naltrindole), and *κ* (nor-BNI) antagonists were administered intrathecally separated by 10 min in cumulative doses to examine whether the effect of EA was blocked by these antagonists. The EA was also applied into Zusanli (ST36). Results show that relieving effects on mechanical allodynia are blocked by *μ* and *δ* selective opioid antagonists but not by *κ* selective opioid antagonists. The fact that *κ* selective opioid antagonist did not work might be due to the low frequency (2 Hz) used in the experiment. Chen and Han [48] and Wu et al. [49] reported that 2 Hz EA-induced analgesia is mediated by met-enkephalin via *μ*, *δ* receptors; however, the antinociception effect induced by high-frequency (100 Hz) EA is mediated by dynorphin via *κ* receptors in the spinal cord of rats. This report is consistent with the review of Han [39].

Kim et al. [30] also reported that the increased expression level of CCK-A receptors, the site of action for the antiopioid peptide cholecystokinin (CCK) in the hypothalamus, might decrease the sensitivity of EA and result in the decrease of the analgesic effect and antiallodynic effect on neuropathic pain model rats. This result is supported by other studies of Lee et al. [50, 51] reporting that the presence of CCK-A receptor might decrease the analgesic effect of EA.

### 2.2. Adrenergic Receptors

Noradrenalin (NE) is known to be one of the main transmitters involved in the descending inhibitory system with serotonin and opioids [43]. It was previously reported that noradrenergic inputs in the spinal cord originate from the locus coeruleus (LC) and adjacent noradrenergic nuclei in the brainstem [52]. Unlike the serotonergic axons descending from nucleus raphe magnus (NRM) and operating through enkephalinergic interneurons in the spinal cord, noradrenergic fibers are known to bring about direct inhibition on the many types of spinal cell with which they make synaptic contacts [53]. Supraspinal descending pathways are known to be the only source of NE in the spinal dorsal horn [54].

There are two major groups of adrenoreceptors, *α* and *β*, with several subtypes. Among these receptors, *α*_1_- and *α*_2_-adrenoreceptors are shown to be largely involved in pain modulation [54], and results from recent studies indicate that both the *α*_1_- and *α*_2_-adrenoreceptors are involved in neuropathic pain [55]. Some articles report that NE enhances the spinal GABAergic and cholinergic transmission by activating *α*_1_- [56] and *α*_2_- [57] adrenoreceptors. An analgesic effect in the rat, caused by intrathecal administered NE, has been shown to be blocked by phentolamine, a nonselective *α*-adrenoreceptors antagonist [43]. Also, epidural injection of the *α*_2_-adrenoreceptors, clonidine, has been reported to result in pain relief in cancer patients with neuropathic pain [58].

To examine the role of *α*_1_- and *α*_2_-adrenoreceptors in the mechanisms of EA, Kim et al. conducted a research administering one dose of *α*_1_- and *α*_2_-adrenoreceptors antagonists (prazosin or yohimbine, resp.) intrathecally with EA. Needles were inserted into Zusanli (ST36), and 30 *μ*g of prazosin and yohimbine were injected to neuropathic pain rats [32]. The relieving effects of EA on cold allodynia were blocked by the *α*_2_-adrenoreceptors antagonist yohimbine but not by the *α*_1_-adrenoreceptors antagonist prazosin. This result shows that the effect of an EA analgesic might be mediated by the spinal *α*_2_-adrenoreceptors but not by the *α*_1_-adrenoreceptors. This data is consistent with Kim's et al. [59] previous study conducted with only *α*_1_- and *α*_2_-adrenoreceptor antagonists, prazosin, and yohimbine intraperitoneally administrated without the EA insertion. Jiang et al. and several other studies [60–63] also reported that intrathecal administration of the *α*_2_-adrenoreceptors agonist in a neuropathic pain model rats significantly attenuated hyperalgesia and tactile allodynia.

Contrary to the role of *α*_2_-adrenoreceptors, a lot of evidence and results from these experiments suggest that not only are spinal *α*_1_-adrenoreceptors not involved in pain inhibition [64, 65] but also they play an important role in the prenociception of animals and humans [66, 67]. These results are in agreement with other previous studies [54, 68, 69] which show how in the nervous system *α*_1_-adrenoreceptors and *α*_2_-adrenoreceptors antagonist yohimbine work as an excitatory although *α*_2_-adrenoreceptors and *α*_1_-adrenoreceptors antagonist prazosin works as an inhibitory and demonstrate that spinal *α*_2_-adrenoreceptors are involved in the relieving effects of EA on cold allodynia.

### 2.3. Serotonergic Receptors

Serotonin is known to have antinociceptive effect spinally, depending on the receptor type activated and dosage use [70, 71]. The role of serotonin in the descending inhibitory pathway of the CNS was also demonstrated [72, 73]. The involvement of serotonin in the analgesic effect of EA was mentioned in an early study of Cheng and Pomeranz [74]. They previously hypothesized that two distinct pain relieving mechanisms are involved in the effects of EA, including endogenous endorphin and nonendorphin systems. They further reported that nonendorphinergic actions may be mediated by monoaminergic neurons such as serotonin and NE [13]. The analgesic effect of serotonin is reported to be mediated from periaqueductal grey (PAG), NRM, and serotonergic receptors present in the spinal dorsal horn [75]. The spinal release of opioid may be driven by a serotonergic descending pathway [76–78] and is at least in part elicited by activation of 5-HT_3_ receptors [79]. The electrolytic lesion of the NRM, a procedure known to decrease the release of 5-HT in the spinal cord [80], attenuates EA-induced analgesia [81]. Research from a number of studies has demonstrated that analgesia by peripheral stimulation is mediated by the serotonergic pathway in the descending inhibitory system [82–84], and the involvement of serotonin receptors in the analgesic mechanism of acupuncture in a neuropathic pain model rat was shown in the study of Zhao [85]. Seven subtypes (5-HT_1–7_) of serotonin receptors have been identified. 5-HT_1_ 5-HT_2_ or 5-HT_3_ are known to be the most commonly implicated in the spinal analgesic effect induced by peripheral stimulation [86–88].

However, conflicting results have been reported regarding the involvement of these three 5-HT receptors. Horiuchi et al. [89] reported that intrathecal administration of 5-HT_1A_ and 5-HT_3_ but not 5-HT_2A_ receptor agonists inhibited thermal hyperalgesia induced by spinal cord injury. Chang et al. [13] are in agreement with Horiuchi et al. and showed that intracerebroventricular administration of 5-HT_1A_ and 5-HT_3_ but not 5-HT_2A_ receptor antagonists blocked the analgesic effect induced by EA. Baek et al. [12] also demonstrated that in the rat model of collagen-induced arthritis the analgesic effect of EA was blocked by intraperitoneal pretreatment of 5-HT_1A_ receptor antagonist and 5-HT_3_ receptor antagonist but not by 5-HT_2_ receptor antagonist. These results suggest that the EA analgesic effect can be mediated by 5-HT_1A_ and 5-HT_3_ receptors but not by a 5-HT_2A_ receptor.

Conversely, Radhakrishnan et al. [88] by administrating different 5-HT subtype antagonist intrathecally showed that spinal 5-HT_2A_ and 5-HT_3_ but not 5-HT_1A_ receptors mediate transcutaneous electrical nerve stimulation (TENS) and induced antihyperalgesia in inflammatory pain model rats. Takagi and Yonehara [90] also reported that intravenously injected 5-HT_1_, except 5-HT_1A_, 5-HT_2_, except 5-HT_2A_, and 5-HT_3_ receptors are involved in EA-induced analgesia.

Thus, to clarify which serotonin receptor is involved in the spinal mechanisms of EA analgesia on neuropathic pain in rats, Kim et al. conducted a further study [32]. Serotonin receptor antagonists of 5-HT_1A_ (NAN-190, 15 *μ*g), 5-HT_2A_ (ketanserin, 30 *μ*g), and 5-HT_3_ (MDL-72222, 12 *μ*g) were injected intrathecally and needles were inserted into Zusanli (ST36). The relieving effect of EA on cold allodynia was blocked by the 5-HT_1A_ antagonist (NAN-190) and by the 5-HT_3_ antagonist (MDL-72222) significantly, but not by the 5-HT_1_ antagonist (ketanserin). This result is consistent with previous studies showing that 5-HT_1A_ receptors [91–93] and 5-HT_3_ receptors [94–96] have antinociceptive roles.

Also, evidence suggests that 5-HT_1A_ receptors inhibit the nociceptive sign in the second-order spinothalamic tract [97], and 5-HT_3_ involves GABAergic, ENKergic (enkephalinergic), and other classes of spinal intrinsic neurons at the spinal level [82, 98–101].

The discordance with the previous result of Radhakrishnan et al. and Takagi and Yonehara might be due to a difference in experiment design, as Radhakrishnan investigated on the inflammation of the knee joint and used TENS but not EA. And Yonehara investigated in the trigeminal nucleus caudalis in rabbits.

### 2.4. Cholinergic Receptors

Cholinergic receptors are known to have both excitatory and inhibitory actions. They mediate acetylcholine (ACh) and induce an analgesic effect by the activation of spinal nicotinic or muscarinic acetylcholine receptors. Both the nicotinic and muscarinic receptors are located in the superficial and deep dorsal horn of the spinal cord where nociceptive information is transmitted and modulated [54, 57, 102]. Cholinergic innervations of the dorsal spinal cord are known to be primarily intrinsic [57], but evidence for cholinergic fibers descending from the brainstem to the spinal cord has also appeared as a result of several studies [103, 104].

The role of nicotinic and muscarinic receptors in the mechanisms of analgesia is known to be different. A large majority of studies indicate that antinociceptive and antiallodynic effects of cholinergic drugs are mediated mainly by the muscarinic receptors but not by the nicotinic receptors [57, 105–107]. Also, previous studies reported that systemic administration of atropine (nonselective muscarinic antagonist) prevented the analgesic effects of EA [12, 108].

To investigate whether spinal nicotinic or muscarinic receptors are involved in the relieving effects of EA on cold and warm allodynia, Park et al. conducted research with intrathecally administered atropine (nonselective muscarinic antagonist) and mecamylamine (nonselective nicotinic antagonist) on neuropathic pain model rats [34]. The relieving effects of EA on both cold allodynia and warm allodynia were completely blocked by atropine but not by mecamylamine. This outcome showed that the antiallodynic effect of EA in neuropathic pain rats is mediated mainly by the muscarinic receptor.

A further study was conducted by Park et al. [34], to determine which muscarine receptor subtype is involved in the antiallodynic action of EA. Currently, five classes of muscarinic receptor have been identified (i.e., M_1–5_). However, the subtypes implicated in the spinal nociceptive transmission and modulations are known to be consisting of M_1_, M_2_, and M_3_ [106, 107, 109]. Pirenzepine (M_1_ muscarinic receptor antagonist), methoctramine (M_2_ muscarinic antagonist), and 4-DAMP (M_3_ muscarinic antagonist) were injected intrathecally on rats, and acupuncture needles were inserted into “Zusanli” (ST36). Among these three antagonists, only pirenzepine (M_1_ muscarinic receptor antagonist) completely blocked the relieving effect of EA on cold allodynia and warm allodynia, whereas methoctramine and 4-DAMP did not. 

Kim et al. [33], with intrathecally administered cholinesterase inhibitor neostigmine, showed that EA has an effect that is equivalent to 0.1 *μ*g of neostigmine on neuropathic pain rats. Neostigmine is known to induce analgesia by mediating spinal muscarinic system and especially at the M_1_ receptor subtype. On the other hand, neostigmine is also known to produce dose-dependent side effects such tremor, writhing action, or urination at doses of 0.3, 1 and 3 *μ*g, in some rats [110, 111]. However, the combination of intrathecal neostigmine (0.1 *μ*g) and EA stimulation produced a synergetic effect lasting more than 80 min., becoming maximal at 20 min., the same as a dose of 0.3 *μ*g of neostigmine, but without side effects. In summary, these results demonstrate that EA stimulation activates spinal M_1_ muscarinic receptors to relieve cold and warm allodynia signs in neuropathic pain rats. This conclusion is in agreement with previous studies showing that M_1_ receptor subtype mediates spinal antinociception and antiallodynia [105, 107, 112–114].

### 2.5. GABAergic Receptors

One of the major inhibitory neuropeptides, GABA is known to be contained in the PAG and plays an important role in the descending pain control pathway of the CNS [115–118]. It is also reported to be involved in multiple physiological and pathological functions. In the spinal cord, GABA exerts tonic modulation of nociceptive neurotransmission between primary afferents and second-order spinothalamic tract neurons [119, 120]. Intrathecally injected baclofen (GABA_B_ receptor agonist) has been demonstrated to produce analgesia in animal models of acute and neuropathic pain [121]. Three GABA receptor subtypes have been identified: GABA_A_, GABA_B_, and GABA_C_ [120], but it has been known that GABA_A_ and GABA_B_ receptors, present in the spinal cord [122], mainly contribute to modulation of pain [54, 123]. Also, GABA_A_ and GABA_B_ receptor agonists have been demonstrated to have antinociceptive effects in a variety of rodent models [124]. The role of GABA and its receptors, in the acupuncture analgesia, has been demonstrated by several studies. Han et al. [125] reported that the microinjecting of muscimol, a GABA_A_ receptor agonist, or 3-MP, a GABA synthesis inhibitor, into the PAG remarkably potentiated or suppressed acupuncture analgesia, respectively. And Fusumada et al. [126] proved that by inserting EA at “Zusanli” (ST36), EA could induce analgesic effect along with the increasing expression of GABA in PAG. Also, Fu and Longhurst [127] and Tjen-A-Looi et al. [128] studies reported that EA decreases the release of GABA in ventrolateral PAG, by modulating the sympathoexcitatory reflex responses through endocannabinoids. These results are in line with the study of Fusumada as the decrease of GABA release may result in the increase of GABA in PAG.

First, Park et al. conducted research to investigate whether spinal GABAergic receptors are involved in the relieving effects of EA on cold allodynia in a rat tail model of neuropathic pain [35]. EA stimulation was applied to “Zusanli” (ST36) and rats were intrathecally injected with gabazine (GABA_A_ receptor antagonist, 0.0003, 0.001, or 0.003 *μ*g) or saclofen (GABA_B_ receptor antagonist, 3, 10, or 30 *μ*g). The relieving effect of EA on cold allodynia on neuropathic pained rats was blocked by gabazine at a dose of 0.001 or 0.003 *μ*g. Saclofen also blocked the effect of EA-induced analgesic effect at a dose of 10 or 30 *μ*g. The results show that both the GABA_A_ and GABA_B_ receptor antagonists dose dependently blocked the relieving effects of EA on cold allodynia. Also, these findings are consistent with the previous studies of Zhu et al. [129, 130], in which intrathecal administration of GABA_A_ and GABA_B_ receptor antagonists partially blocked the acupuncture analgesia. In brief, this evidence supports EA-induced antiallodynia as partially mediated by the activation of spinal inhibitory receptors including GABA receptors. Therefore, it is possible that EA treatment could enhance the analgesic effects of the GABAergic drugs, such as GABA agonists, on neuropathic pain in clinics and vice versa.

## 3. Summary and Discussion

Neuropathic pain is a complex phenomenon, involving several independent pathophysiological mechanisms in both peripheral and CNS. The accurate mechanisms of neuropathic pain and the relationships between its mechanisms, signs, and symptoms are not fully understood, and no consensus on the optimal management of neuropathic pain has been established yet. Although acupuncture's mechanisms of antinociception have not been fully explained, due to the overwhelming amount of research investigated in the last several decades, its analgesic effects are gradually being understood.

**Figure 1:**
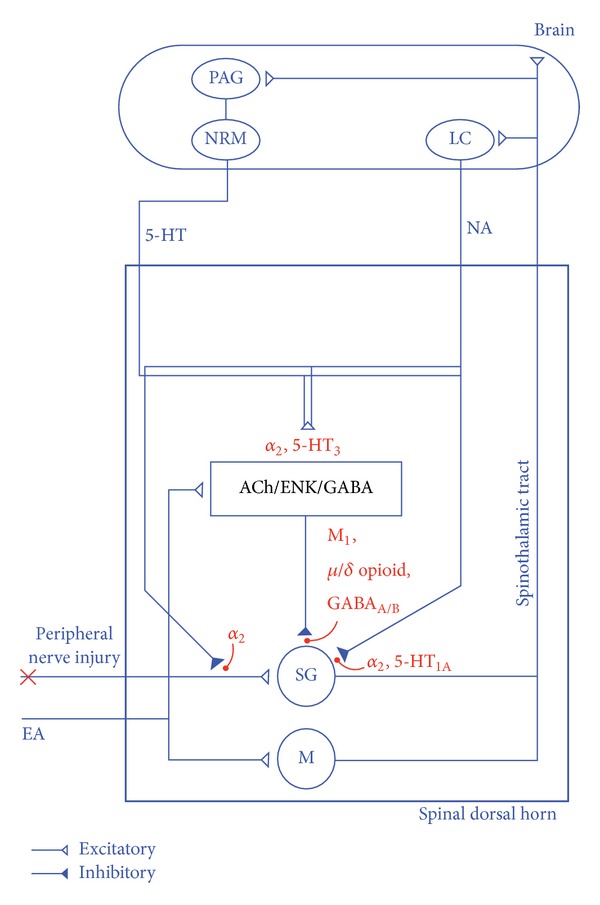
Schematic diagram of EA-induced analgesia in neuropathic pain in the CNS. The peripheral injury information is first transmitted to substantia gelatinosa (SG) cells by primary afferent fibers. EA stimulation is carried up from marginal (M) cells tract to the brain via spinothalamic tract, where the signal is transmitted to the cortex and becomes conscious and also to intrinsic dorsal neurons where it involves cholinergic, ENKergic, and GABAergic neurons. The PAG in the midbrain projects down to the NRM in the middle of the medulla oblongata, and this in turn sends 5-HT fibers to the dorsal horn. LC sends NE fibers to the dorsal horn. NE, via *α*_2_-adrenoreceptors, enhances the spinal cholinergic and GABAergic neurons, involves a reduction in the release of pronociceptive transmitters in the primary afferents fibers, and inhibits the transmission of pain signals to the supraspinal level in the secondary afferents fibers. Serotonin activates enkephalin (ENK) and GABA intrinsic neurons through 5-HT_3_ serotonergic receptors and inhibits secondary afferents fibers via 5-HT_1A_ serotonergic receptors. Cholinergic, ENKergic, GABAergic neurons, through its M_1_ muscarinic receptors, *μ*, *δ* opioid receptors, and GABA_A_ and GABA_B_ receptors control nociceptive inputs from the periphery to higher areas in the CNS. Abbreviations are as follows: SG: substantia gelatinosa; M: marginal cells; PAG: periaqueductal grey; NRM: nucleus raphe magnus; LC: locus coeruleus; 5-HT: serotonin; NE: noradrenalin; Ach: acetylcholine; and ENK: enkephalin.

In this review, based on published reports from several research laboratories around the world and particularly the work performed in our laboratory, we demonstrated that spinal opioidergic, adrenergic, serotonergic, cholinergic, and GABAergic systems mediate the analgesic effects of EA in neuropathic pain rats. Data from our experiments show that spinal *μ* and *δ* opioid receptors, *α*_2_-adrenoreceptors, 5-HT_1A_ and 5-HT_3_ serotonergic receptors, M_1_ muscarinic receptors, and GABA_A_ and GABA_B_ GABAergic receptors are involved in the analgesic effect of EA on neuropathic pain, mediated by the descending inhibitory system in the CNS (Figure 1).

The descending inhibitory pathway consisted of hypothalamus-PAG-rostal ventromedial medulla (RVM)-dorsal horn and mediates the release of serotonin in the PAG and NE in LC. NE, via *α*_2_-adrenoreceptors in the dorsal horn, enhances the spinal cholinergic and GABAergic intrinsic neurons, involves a reduction in the release of pronociceptive transmitters in the primary afferents fibers, and inhibits the transmission of pain signals to the supraspinal level in the secondary afferents fibers [131]. Serotonin activates enkephalin (ENK) and GABA spinal intrinsic neurons through 5-HT_3_ serotonergic receptors and inhibits secondary afferents fiber via 5-HT_1A_ serotonergic receptors [54]. Spinal cholinergic, ENKergic, GABAergic neurons, through its M_1_ muscarinic receptors, *μ*, *δ* opioid receptors, and GABA_A/B_ receptors control nociceptive inputs from the periphery to higher areas in the CNS [54, 57]. The EA stimulation is carried up from marginal (M) cells tract to the brain via spinothalamic tract, where the signal is transmitted to the cortex and becomes conscious, and also to intrinsic dorsal neurons where it involves cholinergic, ENKergic, and GABAergic neurons [53, 121, 132].

Also, the role of glial activation on EA-induced analgesia should be considered alongside the mechanisms of neurons, as microglial activation has been reported to contribute to the initiation of pathological pain responses and astrocytic activation to pain maintenance in a rat model of neuropathic pain [133, 134]. The involvement of glial activation in the analgesic effect of EA is demonstrated in the article of Wang et al. [135], and Gim et al. [36] reported recently that repeated EA attenuates mechanical and warm allodynia by suppressing microglial and astrocyte activation inhibiting the release of proinflammatory cytokine such as TNF-*α*, IL-6, and IL-1*β*.

Most of the articles included in this review used mechanical allodynia and thermal allodynia (warm or cold) to assess the effect of EA. However, they are reported to be mediated differently. Shir and Seltzer [136] demonstrated in his work that mechanical allodynia is mediated by A-fibers and thermal allodynia by C-fibers. Among the works included in this review, Hwang et al. [28] and Kim et al. [29] used mechanical allodynia, while Kim et al. [32, 59], Park et al. [34, 35], and Kim et al. [33] used thermal allodynia to assess the effect of EA on neuropathic pain model rat. On mechanical allodynia the effect of EA marked significant increase up to 20 min after 30 min of EA administration [28], while on thermal allodynia, the EA group showed statistically significant increases in response to latency for up to 50 min after 30 min of EA insertion [32]. These results suggest that the analgesic effect of EA may be more efficacious on thermal allodynia than on mechanical allodynia.

This review is based on articles with animal experience and does not include any controlled clinical trial. Some controlled clinical trials have been published previously to assess the effectiveness of EA on neuropathic pain; however, the data are still controversial, and the number of controlled clinical trials is insufficient to determine its role in the clinic [137, 138]. Although, there are still no randomized clinical trials to support the analgesic effect of EA on neuropathic pain in clinic, we believe that various experimental models and results reported from the work included in our review could guide future efforts to publish a well-designed randomized clinical trial on neuropathic pain. 

## 4. Conclusion

 In conclusion, based on results from our study, we can conclude that both endogenous opioid system and descending inhibitory system mediate the antiallodynic mechanism of EA, and that spinal opioidergic, adrenergic, serotonergic, cholinergic and GABAergic systems are involved in the mechanisms. Also, these results suggest that EA can be an efficacious complementary and alternative treatment to relieve the neuropathic pain. 

Furthermore, since a functional interrelationship exists among the opioidergic, noradrenergic, serotonergic, cholinergic and GABAergic systems in the spinal dorsal horn and supraspinal level [54, 57], further studies should investigate how those receptors interact and to what degree each receptor system contributes independently to EA induced analgesia.
